# The Role of Glycated Hemoglobin A1c in Determining the Severity of Coronary Artery Disease in Diabetic and Non-Diabetic Subjects in Karachi

**DOI:** 10.7759/cureus.4982

**Published:** 2019-06-24

**Authors:** Ibtehaj Ul-Haque, Zia Ud Deen, Shiza Shafique, Syed Inam Ur Rehman, Maryam Zaman, Syeda T Basalat, Misbah Munaf, Yusra Wahidi

**Affiliations:** 1 Internal Medicine, Dow University of Health Sciences (DUHS), Karachi , PAK; 2 Internal Medicine, Dow University of Health Sciences (DUHS), Karachi, PAK; 3 Internal Medicine, Civil Hospital Karachi, Dow University of Health Sciences (DUHS), Karachi, PAK; 4 Miscellaneous, Dow University of Health Sciences (DUHS), Karachi, PAK; 5 Internal Medicine: Critical Care, Dow University of Health Sciences (DUHS), Karachi, PAK

**Keywords:** diabetes, hba1c, non-diabetics, syntax score, coronary artery disease, cardiology, systolic blood pressure, three-vessel disease, endocrine

## Abstract

Introduction

Coronary artery disease is the leading cause of death worldwide by incidence. Over the years, many studies have been conducted to find predictors of coronary artery disease; however, in the last few decades, the level of HbA1c in diabetics has been investigated as a potential predictor. Our study offers additional insight by investigating similar relationships in non-diabetic patients and by investigating potential predictors more comprehensively, making it the first of its kind study. The aim of our study is to indicate that rising HbA1c levels suggest that there’s a greater risk of coronary artery disease, which can further be confirmed by the SYNTAX score, degree of stenosis, and numbers of vessels involved.

Methods

Data from 177 diabetic and 378 non-diabetic patients, all of whom were above 18 years of age, were included in the research. HbA1c levels (>5.6%), SYNTAX score, hypertension, number of vessels involved, and other demographic elements, such as age, smoking, and body measurements, were calculated and compared for diabetics and non-diabetics.

Results

HbA1c was higher in comparison to non-diabetic subjects (p <0.001). Age >53 was found to be a predictor for SYNTAX score ≥23 in diabetic patients (p <0.05). Male gender and smoking were found to be independent predictors for three-vessel disease in the non-diabetic population (p-value<0.05). There was no significant relationship between the SYNTAX score and HbA1c levels in non-diabetics (p=0.885) and diabetics. In conclusion, there is no correlation between elevated HbA1c levels and SYNTAX score ≥23.

## Introduction

Cardiovascular diseases, specifically coronary artery disease (CAD), over the last 10 years has grown to become the world’s largest cause of fatality [1]. The higher incidence and increased risk of developing CAD have been positively linked with diabetes mellitus (DM) in a number of studies [2]. Diabetes mellitus (DM) is a group of metabolic diseases characterized by hyperglycemia resulting from defects in insulin secretion, response to insulin, or both [3]. Glycated haemoglobin (HbA1c) is widely acknowledged as a pivotal and more accurate criterion for pre-empting the severity of DM over fasting glucose levels [4]. HbA1c levels offer more advantage by providing a stable index of long-term glycemic status, which is related to two to three months of average glucose concentration in plasma [5]. Elevated HbA1c levels are notably linked to severe coronary artery atherosclerosis [6-7]. The American Diabetes Association, in its recent position statement, stated that lowering HbA1c may be associated with a reduction in the microvascular, neuropathic, and possibly macrovascular complications of DM [8]. Similarly, since 2004, in non-diabetics, studies linking the relation between the severity of CAD and cardiovascular mortality with HbA1c levels have been widely conducted [5]. Further, they provide evidence that the risk for developing CAD is significantly higher with elevated HbA1c levels even within the normal range [9].

With consistent evidence that CAD and HbA1c levels are predictors of cardiovascular mortality, in this study, we used the SYNTAX score and other variables to compare the severity of coronary artery disease in diabetic and non-diabetic patients in a tertiary care hospital in Karachi, Pakistan.

## Materials and methods

This was a cross-sectional, prospective, and observational study based on a single center. The study was conducted during a time span of 97 days, from January 22, 2018, to April 28, 2018. We randomly selected 177 diabetic patients and 378 non-diabetic patients. All the patients were above the age of 18 years. They had presented to the outpatient department or were admitted to the cardiology ward of Civil Hospital Karachi for clinical reasons, which required them to undergo the coronary angiography procedure. Approval for the protocol of our research was obtained from Pakistan Medical Association Committee (PMA) on Ethics Review Board along with written consent from the cohort involved in our study.

Our exclusion criteria comprised any patient that had already undergone coronary artery bypass, procedures apart from left heart catheterization, and had hemoglobin levels of less than 10.0 gm%.

Hypertension (HTN) and smoking were defined according to the outlines laid by the Joint National Committee in their seventh report on HTN and the interview survey conducted on national health, respectively.

All the patients identified with acute coronary syndrome (ACS) were categorized under unstable angina (USA), non-ST-elevated myocardial infarction (NSTEMI), or ST-elevated myocardial infarction (STEMI), in accordance with the guidelines of the American Heart Association for ACS, 2014. The severity of CAD was graded by cardiologists blinded to the levels of HbA1c [10]. The SYNTAX score was applied for the calculations and anyone with a score of >22 was established as a case of severe CAD [11].

We obtained samples of patients for fasting plasma glucose and HbA1c while they were in their overnight fasting state prior to the procedure and classified them into the following divisions based on their HbA1c: Non-Diabetics with HbA1c<5.1, Pre-Diabetics with HbA1c 5.1-6.4, and Diabetics with HbA1c>6.4. An exchange chromatography method was used for discerning the levels of HbA1c. We considered all the demographic elements such as age, sex, ethnicity, risk factors predisposing to CAD, and varying body measurements. Depending on the patient's choice and accessibility to test at the time, a left ventricular angiogram or echo was used to assess the ejection fraction (EF) of the left ventricle and all angiographic scans were evaluated by well-reputed cardiac specialists.

If any of the major vessels or their branches were found to have more than 50% stenosis, we designated it as significant CAD and categorized it as follows: Obstructed with ≥70-100% stenosis or Non-Obstructed with >50% and <70% stenosis. Patients were labeled as having either single vessel disease (SVD), double vessel disease (DVD), or triple vessel disease (TVD) based on the number of arteries involved from the three major coronary vessels, namely, Left Anterior Descending, Left Circumflex Artery, and Right Coronary Artery.

The primary incentive was to estimate the relationship of HbA1c values with the severity of CAD.

## Results

In our study, 177 patients had diabetes (D) while 378 patients were non-diabetic (N-D). The majority of the patients, 79.1% of non-diabetics and 71.2% of diabetics, were males. The clinical, laboratory, and cardiac investigations of study subjects are mentioned in Table 1. The results of several clinical and laboratory investigations were found to have strong statistical significance. Diabetic patients had a greater incidence of HTN, with a strong statistically significant difference between the two groups (p<0.001, D=60.4%, N-D=43.1%). HbA1c levels were higher in diabetic patients with a strong statistical difference between the two groups (p<0.001, D= 8.7±2.07, N-D=6.0 ± 1.21). Most diabetic patients had a body mass index (BMI) of more than 25 kg/m^2^, the few that were below 25 kg/m^2^ revealed statistically significant differences from the non-diabetic group (p=0.002, D(<25 kg/m^2)^= 39.8%, N-D(<25 kg/m^2)^= 50.6%). Mean waist circumference was slightly greater in diabetic patients with a slight statistically significant difference from the non-diabetic group (p=0.002, D=39.6± 5.03, N-D=38.2 ± 5.51). Mean systolic blood pressure (SBP) was higher in the diabetic group with a slight statistically significant difference between the groups (p=0.023, D=127 ± 17.8, N-D=123±19.8).

**Table 1:** Comparison between diabetics and non-diabetics S.D: standard deviation; HTN: hypertension; SBP: systolic blood pressure; DBP: diastolic blood pressure; BMI: body mass index; CAD: coronary artery disease; SVD: single vessel disease; DVD: double vessel disease; TVD: triple vessel disease; ACS: acute coronary syndrome; USA: unstable angina; NSTEMI: non-ST-elevated myocardial infarction; STEMI: ST-elevated myocardial infarction; EF: ejection fraction

S. No.		Non-Diabetics (n=378)	Diabetics (n=177)	p-value
1	Age in years (Mean ± S.D)	53.0 ± 11.21	54.4 ± 9.38	0.164
2	Gender			
	Male	299 (79.1%)	126 (71.2%)	0.052
	Female	79 (20.9%)	51 (28.8%)	
3	HTN	163 (43.1%)	107 (60.4%)	0.001
4	Smoker	170 (44.9%)	64 (36.2%)	0.050
5	SBP at admission (Mean ± S.D)	123 ± 19.8	127 ± 17.8	0.023
6	DBP at admission (Mean ± S.D)	80 ± 12.6	82 ± 10.1	0.057
7	HbA1c (Mean ± S.D)	6.0 ± 1.21	8.7 ± 2.07	0.001
8	Glucose (mg/dl) (Mean ± S.D)	120 ± 60	114 ± 23	0.001
9	BMI	n=350	n=161	
	≤ 25	177 (50.6%)	64 (39.8%)	0.002
	> 25 – 30	117 (33.4%)	67 (41.6%)	0.073
	> 30	56 (16.0%)	30 (18.6%)	0.459
	Mean ± S.D	25.2 ± 4.98	26.7 ± 5.19	0.002
	Waist Circumference (Mean ± S.D)	38.2 ± 5.51	39.6± 5.03	0.010
	Waist /Hip Ratio (Mean ± S.D)	0.98 ± 0.06	0.98 ± 0.05	0.947
10	SYNTAX Score	n=160	n=79	
	≤ 22	119 (74.4%)	50 (63.3%)	0.007
	≥ 23	41 (25.6%)	29 (36.7%)	
	Mean ± S.D	16.1 ± 10.18	20.1 ± 10.47	0.005
11	CAD	n=236	n=112	
	Normal	32 (13.6%)	9 (8.0%)	0.135
	SVD	80 (33.9%)	21 (18.8%)	0.003
	DVD	54 (22.9%)	21 (18.8%)	0.381
	TVD	70 (29.7%)	61 (54.5%)	0.001
	Obstruction	n=204	n=112	
	Non obstructed	46 (22.5%)	13 (12.6%)	0.037
	Obstructed	158 (77.4%)	90 (87.4%)	
12	Types of ACS	n=370	n=168	
	USA	120 (33.4%)	61 (36.3%)	0.469
	NSTEMI	161 (43.5%)	61 (36.3%)	
	STEMI	89 (24.1%)	46 (27.4%)	
13	EF % (Mean ± S.D)	49.7 ± 16.51	51.5 ± 15.37	0.576

Cardiac investigations yielded only a few significant results. The SYNTAX score could only be calculated in 79 diabetic patients and 160 non-diabetic patients. A SYNTAX score of equal to or greater than 23 was obtained in 36.7% and 25.6% of diabetic and non-diabetic patients with a significant difference between the mean score of two groups (p=0.005, D=20.1±10.47, N-D=16.1 ± 10.18). In CAD, the incidence of triple vessel disease was marked in diabetic patients with a strongly significant difference from non-diabetic patients (p<0.001, D=54.5%, N-D=29.7%) while for single vessel disease, the incidence was greater in non-diabetic patients with a slightly significant difference from the diabetic group (p=0.003, D=18.8%, N-D=33.9%). Vessel obstruction was found more commonly in diabetic patients with a slight statistically significant difference from the non-diabetic group (p=0.037, D=87.4%, N-D=77.4%). The remaining cardiac investigations remained statistically insignificant.

Multivariate logistic regression was calculated for non-diabetic (see Table 2) and diabetic patients (see Table 3) with SYNTAX score ≥23 (including male gender, age ≥53 years, HTN, smoking, BMI>25, and HbA1c >6.4 as dependent variables). None of the variables were found to be independent predictors for SYNTAX score ≥23 in the non-diabetic population while age >53 years was an independent predictor for SYNTAX score ≥23 in the diabetic population (Beta (age) = 1.432, p <0.05).

**Table 2:** Multivariate logistic regression in non-diabetics B: beta; S.E: standard error; Sig.: significance; Exp(B): exponential for beta coefficient; C.I: confidence interval; BMI: body mass index

SYNTAX Score ≥ 23	B	S.E.	Sig.	Exp(B)	95% C.I for EXP(B)	
					Lower	Upper
Male	-.074	.626	.905	.928	.272	3.168
Age >53	.227	.445	.610	1.254	.524	3.002
Hypertension	-.389	.474	.411	.677	.268	1.714
Smoking	.453	.503	.367	1.573	.587	4.216
BMI >25	.044	.446	.922	1.045	.436	2.505
HbA1c >6.4	.085	.587	.885	1.088	.345	3.436
3-Vessel disease	-1.953	.455	.000	.142	.058	.346

**Table 3:** Multivariate logistic regression in diabetics B: beta; S.E: standard error; Sig.: significance; Exp(B): exponential for beta coefficient; C.I: confidence interval; BMI: body mass index

SYNTAX Score ≥23	B	S.E.	Sig.	Exp(B)	95.0% C.I. for EXP(B)	
					Lower	Upper
Male	-.495	.737	.502	.610	.144	2.583
Age >53	1.432	.694	.039	4.189	1.074	16.333
Hypertension	-.432	.698	.536	.649	.165	2.549
Smoker	-.932	.668	.163	.394	.106	1.458
BMI > 25	.091	.696	.896	1.096	.280	4.285
HbA1c > 6.4	-.878	.840	.296	.416	.080	2.156
3-Vessel disease	2.290	.841	.006	9.879	1.902	51.305

Multivariate logistic regression for non-diabetics (see Table 4) and diabetic patients (see Table 5) with three-vessel disease was calculated (including male gender, age ≥53, HTN, smoking, BMI >25, and HbA1c >6.4 as dependent variables). Male gender and smoking were found to be independent predictors for three-vessel disease in the non-diabetic population (Beta (male) = 1.446, Beta (smoking) = -1.268, p-value<0.05), whereas no variable was seen to be an independent predictor for 3-Vessel diseases in diabetic population (Beta = 2.324, p<0.05).

**Table 4:** Multivariate logistic regression in non-diabetics B: beta; S.E: standard error; Sig.: significance; Exp(B): exponential for beta coefficient; C.I: confidence interval; BMI: body mass index

3-Vessel disease	B	S.E.	Sig.	Exp(B)	95% C.I. for EXP(B)	
					Lower	Upper
Male	1.446	.621	.020	4.246	1.258	14.333
Age >53	-.538	.434	.215	.584	.250	1.367
Hypertension	.230	.456	.615	1.258	.514	3.079
Smoking	-1.268	.476	.008	.281	.111	.715
BMI >25	-.101	.444	.820	.904	.379	2.157
HbA1c > 6.4	-.913	.568	.108	.401	.132	1.223
SYNTAX Score ≥23	-1.957	.456	.000	.141	.058	.345

**Table 5:** Multivariate logistic regression in diabetics B: beta; S.E: standard error; Sig.: significance; Exp(B): exponential for beta coefficient; C.I: confidence interval; BMI: body mass index

3-Vessel disease	B	S.E.	Sig.	Exp(B)	95.0% C.I. for EXP(B)	
					Lower	Upper
Male	-.133	.751	.860	.876	.201	3.815
Age > 53	.121	.637	.850	1.128	.324	3.934
Hypertension	.910	.632	.150	2.485	.719	8.584
Smoker	-.132	.639	.836	.876	.250	3.067
BMI > 25	.800	.634	.207	2.225	.643	7.708
HbA1c > 6.4	.156	.787	.843	1.169	.250	5.468
SYNTAX Score ≥23	2.324	.863	.007	10.215	1.882	55.451

## Discussion

Our study, to the best of our knowledge, is one of the first few studies from Karachi, Pakistan, in which the role of HbA1c is determined for assessing the severity of CAD in diabetic and non-diabetic subjects using the SYNTAX score. In our study, elevated HbA1c did not correlate with the number of vessels involved and the SYNTAX score. The trend showed that, on average, the SYNTAX score was higher in diabetics as compared to non-diabetics. Interestingly, single vessel disease had a greater occurrence in non-diabetics whereas triple vessel disease was more common in diabetics. Subjects were found to have significantly higher HbA1c levels as compared to non-diabetic subjects. Accordingly, the incidence of triple vessel disease and vessel obstructions was significantly higher in diabetic subjects as compared to non-diabetics. A higher incidence of hypertension was also found among diabetic subjects.

Other general clinical and laboratory investigations, such as fasting blood sugar (FBS) and mean systolic blood pressure (SBP), were also measured. Interestingly, FBS levels were lower in diabetic subjects. Mean SBP was also higher in diabetic subjects compared to non-diabetics.

 In contrast to our study, Kapil et al. revealed a positive predictor relationship between HbA1c and cardiovascular disease (p=0.001) [1]. Moreover, another study from North Eastern India, which used the SYNTAX score also reported a highly significant correlation between elevated HbA1c and higher SYNTAX score [12].

We believe that the significant relationship between male gender and smoking with a three-vessel disease in non-diabetic subjects may emphasize a significant mortality rate, which is comparable to several studies that employed HbA1c as a strong indicator of mortality [13] as well as major cardiovascular complications and target vessel revascularization in non-diabetics [14-15]. One of the studies concluded that smoking was a major factor in the mortality of non-diabetic individuals (p<0.01) [16]. On the contrary, recent studies have shown that regular advances in cardiovascular medicine, specifically the treatment of acute coronary syndrome (ACS), have contributed to the reduction in morbidity and mortality for acute myocardial infarction (MI) and DM [17].

Most of the studies on this subject employed the Gensini score to determine the severity of CAD while we used the relatively recent and less commonly used scoring modality, the SYNTAX score. The Gensini score determines the degree of stenosis in a vessel as compared to the extent of vessel involvement, which is measured by the SYNTAX score. Although we were not able to produce the desired results from this study, this does not demerit the SYNTAX score; instead, it encourages more medical professionals to conduct further studies to collect more data in order to improve our understanding about the application of this test as outlying results are fairly common for any debatable test.

Similar research that was conducted at the Sanjay Gandhi Postgraduate Institute of Medical Sciences, Lucknow, screened 905 patients with CAD, and their results strongly correlated disease severity and higher SYNTAX score (≥23) with elevated HbA1c levels in diabetics [18]. However, our study was not able to provide a positive correlation between the above-mentioned variables. This difference may be due to the limitations of our study, which didn’t include the ongoing drug regimen and any previous intervention.

Our research had several limitations. First, our study period was comparatively short. We believe that this provided an inaccurate representation of HbA1c levels, which was further complicated by unavailable information on the duration of diabetes, previous surgeries, and previous/current drug regimen. This might be a major cause as to why HbA1c was a poor predictor in our study. Second, it was not possible to further follow up on our patients; this, too, prevented us from ascertaining the duration of diabetes and other influencing factors.

Reflecting on our shortcomings and based upon the literature search, certain necessary suggestions for fellow researchers are mentioned below:

- The duration of diabetes should be investigated, as this was found to predict a greater level of coronary vessel occlusions [2].

- Drug regimen and surgical interventions should be included in future studies since these may alter the distribution of coronary lesions, maybe even localizing them. This can potentially decrease the SYNTAX score and provide a false relationship with other variables like HbA1c.

- Additional markers, such as coronary intima-media thickening, glomerular filtration rate (GFR), microalbuminuria, and serum creatinine, should be considered, as they have been successful in pointing toward severe coronary diseases. Carotid intima-media thickness (CIMT) is considered a marker for early atherosclerosis disease, allowing detection in the subclinical stage [1]. Further, microalbuminuria is found to be a strong predictor of the presence and severity of CAD [2].

## Conclusions

The above study suggests that although HbA1c was significantly greater in diabetic patients as compared to non-diabetic patients, this did not positively correlate with the SYNTAX score. This, therefore, demands further investigation into the SYNTAX score as a modality for predicting the severity of coronary artery disease.
